# The *hsp 16* Gene of the Probiotic *Lactobacillus acidophilus* Is Differently Regulated by Salt, High Temperature and Acidic Stresses, as Revealed by Reverse Transcription Quantitative PCR (qRT-PCR) Analysis

**DOI:** 10.3390/ijms12085390

**Published:** 2011-08-22

**Authors:** Vittorio Capozzi, Mattia Pia Arena, Elisabetta Crisetti, Giuseppe Spano, Daniela Fiocco

**Affiliations:** 1 Department of Food Science, University of Foggia, via Napoli 25, 71122 Foggia, Italy; E-Mails: vittorio.capozzi@gmail.com (V.C.); mp.arena@yahoo.it (M.P.A.); 2 ISZ Istituto Zooprofilattico della Puglia e della Basilicata/ via Manfredonia 20, 71121 Foggia, Italy; E-Mail: e.crisetti@izfg.it; 3 Department of Biomedical Science, University of Foggia/ via L. Pinto, 71122 Foggia, Italy; E-Mail: d.fiocco@unifg.it

**Keywords:** RT-qPCR, *Lactobacillus acidophilus*, probiotic, small heat shock gene, stress

## Abstract

Small heat shock proteins (sHsps) are ubiquitous conserved chaperone-like proteins involved in cellular proteins protection under stressful conditions. In this study, a reverse transcription quantitative PCR (RT-qPCR) procedure was developed and used to quantify the transcript level of a small heat shock gene (*shs*) in the probiotic bacterium *Lactobacillus acidophilus* NCFM, under stress conditions such as heat (45 °C and 53 °C), bile (0.3% w/v), hyperosmosis (1 M and 2.5 M NaCl), and low pH value (pH 4). The *shs* gene of *L. acidophilus* NCFM was induced by salt, high temperature and acidic stress, while repression was observed upon bile stress. Analysis of the 5′ noncoding region of the *hsp16* gene reveals the presence of an inverted repeat (IR) sequence (TTAGCACTC-N9-GAGTGCTAA) homologue to the controlling IR of chaperone expression (CIRCE) elements found in the upstream regulatory region of Gram-positive heat shock operons, suggesting that the *hsp16* gene of *L. acidophilus* might be transcriptionally controlled by HrcA. In addition, the alignment of several small heat shock proteins identified so far in lactic acid bacteria, reveals that the Hsp16 of *L. acidophilus* exhibits a strong evolutionary relationship with members of the *Lactobacillus acidophilus* group.

## 1. Introduction

During the last decade the use of microorganisms considered probiotic (health promoting) has increased markedly. Specifically, some lactic acid bacteria (LAB) have been shown to confer some beneficial health effects on the human host. The selection criteria for probiotic microorganisms includes (i) safety, (ii) functionality (e.g., survival, adherence, colonization), and (iii) technological features (e.g., sensory properties, growth, stability, viability during manufacture) [1]. In food matrices and during the gastrointestinal transit, these bacteria are exposed to various kinds of stress conditions, including temperature, acid, bile exposure, and osmotic stress. Naturally, in order to be effective probiotics or vaccine-delivery vehicles, they have to first survive in these complex, harsh environments [2]. However, probiotic bacteria detain complex molecular mechanisms to cope with the often lethal environmental stresses encountered during food processing and following ingestion [3]. A comprehensive assessment of these mechanisms could enhance design and manufacture of probiotic cultures, and help to achieve greater viability during passage along the gastro-intestinal tract [3]. *Lactobacillus acidophilus* is a homo-fermentative LAB species boasting biotechnological applications (i) in the production of dairy foods and (ii) as probiotic (e.g., in the form of yogurts, dietary supplements). It is also being considered as a potential vaccine-delivery vehicle to the gastrointestinal tract [4]. The probiotic properties of *L. acidophilus* comprise balancing of the intestinal microflora, treatment of acute infectious diarrhea, antibiotic-associated diarrhea, and diarrhea-predominant irritable bowel syndrome, cholesterol reduction, decrease of oral streptococci cariogenic potential and alleviation of Crohn’s disease [4–9]. Because of the importance of this organism as probiotic, studies on its stress response mechanisms may be useful in selecting or improving *L. acidophilus* strains able to grow under harsh stress conditions.

Small heat shock proteins (sHsps) are ATP-independent chaperons, whose function is to mediate the correct protein folding in the context of a multi-chaperone network [10]. They act as one of the first biological machinery that copes with stress-induced cell damage by binding and maintaining denatured proteins in a disaggregation-competent state [10]. sHsps are characterized by a conserved α-crystallin domain that is preceded by an N-terminal region of variable length and sequence and followed by a short C-terminal extension. In vitro, they can prevent irreversible protein aggregation by forming soluble oligomeric complex with nonnative proteins [11]. sHsps proteins are induced in response to various kinds of abiotic stress including heat shock, acid stress, and osmotic stress, although some sHsps are also expressed constitutively, under physiological conditions [12,13]. Therefore, they are also usually considered “general” stress proteins. Interestingly, the number of *shsp* genes appears to vary considerably among bacterial species [14,15]. Among them, *L. acidophilus* NCFM genome encodes only one small heat shock protein with a predicted molecular mass of 16.16 kDa [16]. In this work, we report our observations on the expression of the small heat shock gene (*shs*) of *L. acidophilus* NCFM under abiotic stress such as high temperature, acidic, salt and bile stress. We have focused on these specific stress conditions as they are often encountered by LAB either during food fermentation or gastrointestinal transit.

## 2. Results and Discussion

### 2.1. Genomic Organization Lactobacillus Acidophilus hsp16

**Figure 1.**
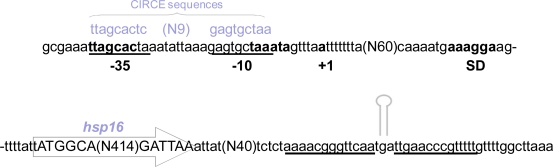
Analysis of the 5′ and 3′ noncoding regions of the *hsp16 locus* in *L. acidophilus* NCFM. In the upstream region: the ORF is encased in an arrow; putative transcription start (+1), −35 and −10 boxes and Shine-Dalgarno sequence are given in bold typeface; horizontal bars represent the controlling IR of chaperone expression (CIRCE) elements. In the downstream region, horizontal dotted lines indicate the position of a putative transcription terminator.

The genomic organization of the *L. acidophilus* NCFM *hsp16* gene is reported in Figure 1. A putative transcription initiation site was mapped to position −90, relative to the translational start codon (ATG). A typical prokaryotic Shine-Dalgarno ribosome binding site (RBS), AAAGGA, is present, complementary to the 3′-end (TCCTTT) of *L. acidophilus* NCFM 16S rRNA. The −10 (TAAATA) and −35 (TTAGCA) boxes, separated by 18 nucleotides, were identified at an appropriate distance from the transcriptional start site. An inspection of the 3′-side noncoding region of the small heat shock gene revealed an inverted-repeat sequence that could form a stem-and-loop structure in the mRNA and it is likely to function as a transcriptional terminator. The proposed transcription start site is preceded by a sequence that shows 63% identity with the extended −10 box consensus sequence (TNTGNTATAAT) of the σA promoters of Gram-positive bacteria [17]. Analysis of the 5′ noncoding region reveals the presence of an inverted repeat (IR) sequence (TTAGCACTC-N9-GAGTGCTAA) homologue to the controlling IR of chaperone expression (CIRCE) elements found in the upstream regulatory region of Gram-positive heat shock operons, suggesting that the *hsp16* gene of *L. acidophilus* might be transcriptionally controlled by HrcA [18].

**Figure 2.**
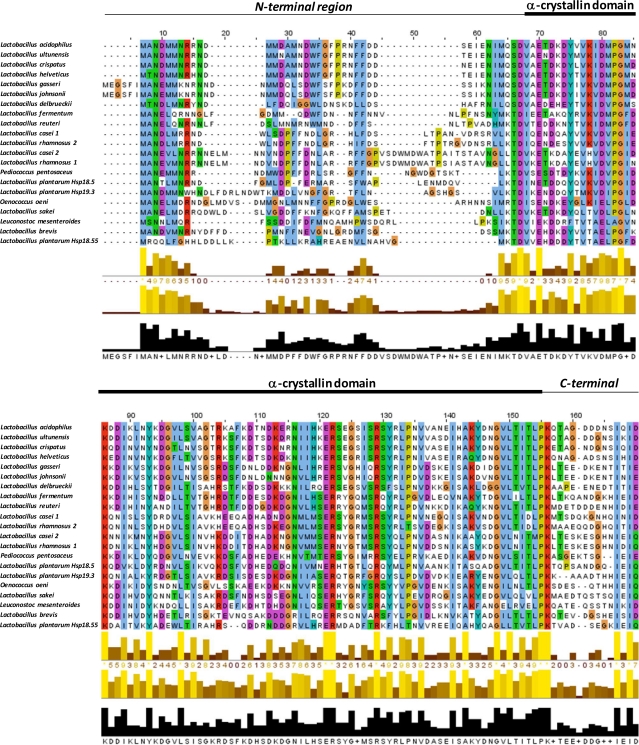
Alignment of the amino acid sequences for Hsp16 from *L. acidophilus* NCFM with other prokaryotic sHsps. The α-crystallin domain and C-terminal and N-terminal regions are indicated. Color shading indicates conservation at a given position of the protein in the alignment.

In order to assess the distribution of sHsp homologs across prokaryotics, especially LAB, we surveyed representative available sequenced genomes for the presence of sHsp-encoding genes (Table 1). Alignment of the sHSPs amino acid sequences indicates moderate similarity among the α-crystallin domains, whereas N and C terminal regions were found to be much more variable (Figure 2).

**Figure 3.**
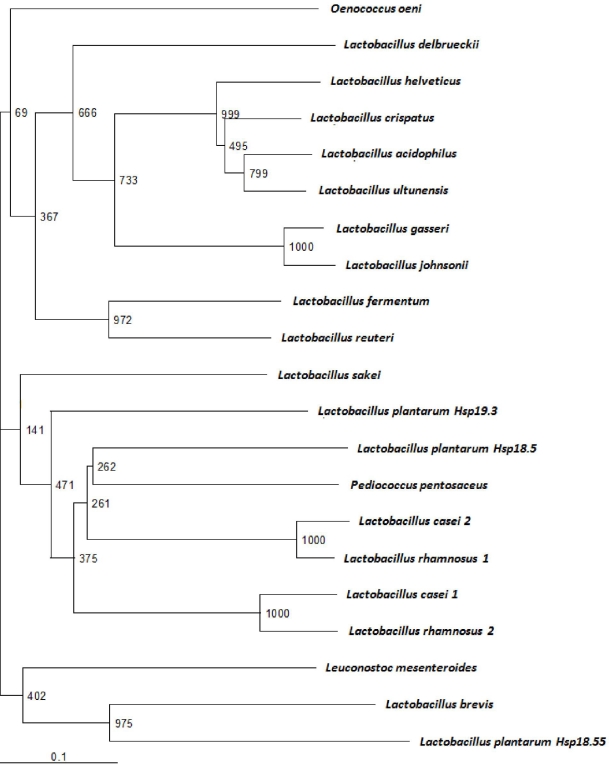
Phylogenetic tree obtained using the α-crystallin domain sHSP protein sequences. Bootstrap values are reported for a total of 1,000 replicates.

Hsp16 exhibits the highest level of sequence identity (90%) with *Lactobacillus ultunensis* sHsp, followed by *Lactobacillus crispatus* sHsp (87%), *Lactobacillus helveticus* sHsp (82%), *Lactobacillus gasseri* sHsp (63%), and *Lactobacillus johnsonii* sHsp (62%). α-crystallin domain protein alignment was performed using ClustalW and resulted in an unrooted neighbor-joining phylogenetic tree (Figure 3). Hsp16 branches together with the sHSP sequences of members of the *Lactobacillus acidophilus* group. More generally, as observed by Ventura *et al.* [14], the distribution of sHsp-encoding genes is expected to be a consequence of either a vertical or horizontal transfer mechanism.

**Figure 4.**
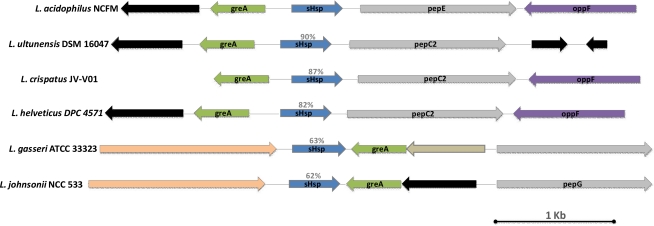
Comparison of the *hsp16 locus* in *L. acidophilus* NCFM with corresponding *loci* in various other bacteria. Each arrow indicates an ORF. The length of the arrow is proportional to the length of the predicted ORF. Corresponding genes are marked with the same color. The putative function of the proteins is indicated above each arrow, and black arrows indicate gene coding for hypothetical proteins. Amino acid identity is shown as a percentage. The ORF name or the *locus* identification (ID) numbers are given.

The *hsp16 locus* in *L. acidophilus* and other bacteria is schematically reported in Figure 4. The comparative analysis was performed with *loci* of the most similar protein to the predicted *L. acidophilus* Hsp16. Generally, in contrast to high molecular weight chaperone members, sHsps show huge variation in sequence, polypeptide size, and oligomer subunit number [12]. To our surprise, in some cases we noticed a considerable homology coupled with a strongly conserved genomic organization of the *shsp loci*. Quite the opposite result was reported by Ventura *et al.* [14] who, studying *hsp20* in *Bifidobacterium breve*, found significant DNA sequence homology detectable between the various *Bifidobacterium* sHsp-encoding genes. However, in contrast, they evidenced a variable organization of the flanking genes.

### 2.2. Relative Expression Levels of hsp16 Gene Under Different Abiotic Stresses

**Figure 5.**
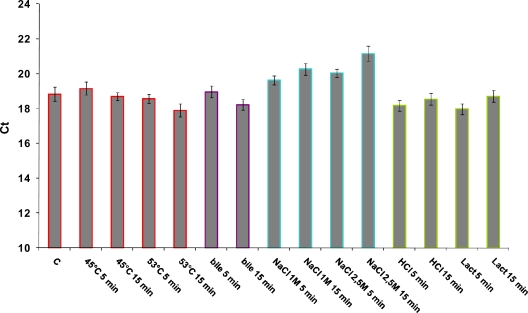
Cycle threshold (CT) of a potential housekeeping gene (*ldhD*) analyzed by reverse transcription quantitative polymerase chain reaction (RT-qPCR). For each condition, CT was measured from three independent cDNAs; the means are represented in the histogram with their standard deviations indicated by vertical bars.

Expression of the *hsp16* gene of *L. acidophilus* was monitored by quantitative Real Time (qRT) PCR in presence of abiotic stresses such as high temperature (45 °C and 53 °C), high salt content (NaCl 1 M and 2.5 M), acid stress (pH 4), presence of bile 0.3% (w/v), or ethanol (12%). All stress conditions were imposed for 5 min and 15 min, and stress intensities were chosen based on previous works [4]. We assessed the use of the *ldhD* gene as internal control for reverse transcription quantitative polymerase chain reaction (RT-qPCR) analysis in *L. acidophilus*. The *ldhD* transcript level was partially affected by the stress conditions tested in our work (Figure 5). In particular, significant difference was observed upon severe heat (53 °C) and bile stresses, suggesting that the identification of a gene as internal control requires further studies. As a consequence, relative gene expressions results were normalized to the quantity of total RNA. *hsp16* expression was calculated relative to the control unstressed cells.

**Figure 6.**
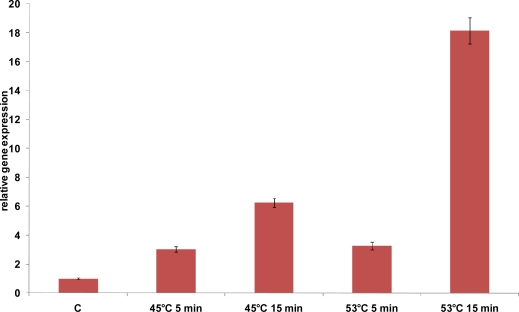
Relative gene expression of *hsp16* of *L. acidophilus* NCFM under heat stress condition as determined by qRT-PCR analysis. mRNA levels were calculated relative to the transcript level detected in control unstressed cultures and were normalized to total RNA content. RNA was extracted and analyzed 5 and 15 min and after exposure to 45 °C and 53 °C. The data presented are the mean of three independent experiments with their standard deviations indicated by vertical bars.

An increased expression level was observed for the *hsp16* gene of *L. acidophilus* either at 45 °C or at 53 °C (Figure 6). A transcriptional induction was already evident after 5 min stress, with a stronger induction after 15 min temperature upshift to 45 °C or 53 °C (7- and 18-fold, respectively) (Figure 6). *hsp16* gene involvement in the response to heat stress is corroborated by the increased gene expression after exposure to 53 °C; indeed, this temperature, identified as sub-lethal, probably represents a significant obstacle to the growth of bacteria cells. The strong induction observed after 15 min at 53 °C may contributes to explain the thermotolerance enhancement revealed by Kim *et al.* [4] after exposure at the same temperature.

**Figure 7.**
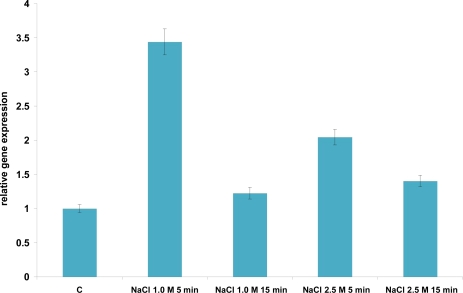
Relative gene expression of *hsp16* of *L. acidophilus* NCFM under osmotic stress conditions, as determined by qRT-PCR. mRNA levels were calculated relative to the transcript level detected in control unstressed cultures and were normalized to total RNA content. RNA was extracted and analyzed 5 and 15 min and after exposure to 1 M and 2.5 M NaCl. The data presented are the mean of three independent experiments with their standard deviations indicated by vertical bars.

**Figure 8.**
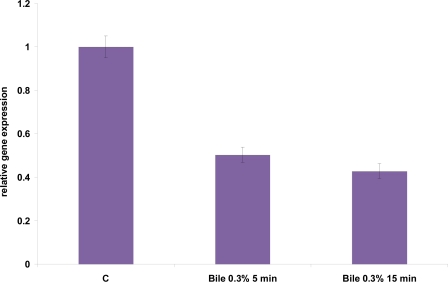
Relative gene expression of *hsp16* of *L. acidophilus* NCFM under bile stress condition, as determined by qRT-PCR. mRNA levels were calculated relative to the transcript level detected in control unstressed cultures and were normalized to total RNA content. RNA was extracted and analyzed 5 and 15 min and after exposure to 0.3% bile. The data presented are the mean of three independent experiments with their standard deviations indicated by vertical bars.

Kim *et al.* [4] previously identified NaCl at 2% and 18% w/v as a sublethal and lethal osmotic stress respectively, allowing growth of *L. acidophilus* cells. Therefore, we used NaCl at the concentration of 1 M (5.8% w/v) and 2.5 M (14.5% w/v), two different values of sublethal salt stress. Hyperosmotic stress transiently induced hsp16 gene expression (Figure 7). Indeed, a maximum increase in the amount of the corresponding mRNA was observed after 5 minutes salt stress with 3- and 2-fold induction when salt was added at the final concentration 1 M and 2.5 M, respectively. Thereafter, a decrease in mRNA level occurred after 15 min stresses were imposed. The adaptive response to NaCl stress was previously shown to provide cross-protection against heat stress [4], but not vice versa, suggesting that at least a subset of heat shock proteins might be as well induced by salt stress, but a temperature upshift may not necessarily induce NaCl stress response. Our results support the hypothesis that a cross-talk does exist between salt and heat response. Indeed, Kim *et al.* [4] observed that the cells pre-exposed to the NaCl stress were significantly more resistant when subjected to heat stress. Similar behavior was observed among LAB in *Lactococcus lactis*, where heat shock proteins DnaK, GroEL, and GroES were induced by salt stress [19]. Given that the liver secretes as much as a liter of bile into the intestinal tract each day, in order to emulsify and solubilize lipids, exposure to bile also represents a harsh challenge for bacteria [20]. Several studies indicate that the molecular chaperones DnaK and GroEL are induced by bile [21–24]. Kim *et al.* [4] also highlighted in previous studies that the cells pre-exposed to the bile stress gained greater resistance to heat stress; conversely, pre-exposure to heat stress could not increase resistance against lethal bile stress. Kim *et al.* [4] identified bile at 0.05% (w/v) as the sublethal level, since cells were still growing slowly at this level, and bile at 0.5% (w/v) as the lethal level, even if not all cells were killed at this level. Therefore, we performed our experiments using bile at 0.3% (w/v), a value that is halfway between the sublethal and lethal level. A low repression of *hsp16* gene (0.5-fold) was detected in response to bile stresses, both after 5 and 15 minutes exposure (Figure 8). This result suggests that Hsp16 is not directly involved in response to stress induced by bile treatment and is not part of the hypothesized cross response to different stress types.

**Figure 9.**
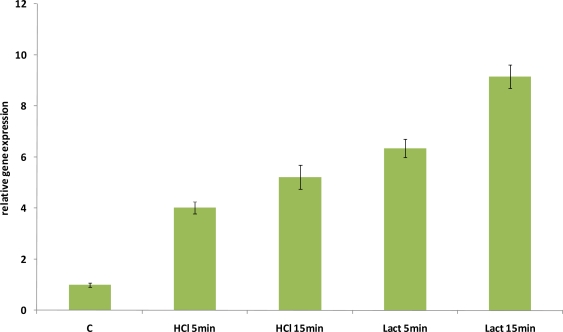
Relative gene expression of *hsp16* of *L. acidophilus* NCFM under acidic stress conditions, as determined by qRT-PCR. mRNA levels were calculated relative to the transcript level detected in control unstressed cultures and were normalized to total RNA content. RNA was extracted and analyzed 5 and 15 min and after exposure to pH 4, obtained by adding either lactic acid or hydrochloric acid. The data presented are the mean of three independent experiments with their standard deviations indicated by vertical bars.

Lactobacilli, including *L. acidophilus,* can compete for growth with other food-borne microbes by taking advantage from the environmental acidification, resulting from their metabolic activities. For this reason, the biological mechanisms allowing LAB to cope with acidic stress have been receiving growing attention. The survival of *L. acidophilus* in acidic environments has been studied, and this species was proven to be highly resistant to acid [25]. Lorca *et al.* [26] found that the heat shock proteins DnaK, DnaJ, GrpE, GroES and GroEL were among those proteins whose synthesis was induced in response to acid adaptation. We investigated the relative hsp16 gene expression during acid stresses (pH 4) obtained by addition of either hydrochloric acid or lactic acid. As shown in Figure 9, *hsp16* gene expression increased when acidic stress was imposed. However, a different level of induction was observed for the two acidifying agents. The highest induction was reported after 15 min (9-fold) of exposure to medium acidified with lactic acid, while a less pronounced induction was detected in presence of hydrochloric acid after 5 (4-fold) and 15 min (5-fold) and in the presence of lactic acid after 5 minutes (6-fold) (Figure 9). Interestingly, not only did we notice a remarkable *hsp16* induction under acidic condition, but we also detected a different pattern in response to the same hydrogen ion concentration, achieved by hydrochloric acid and lactic acid. A possible explanation for these differences could be connected with biological mechanisms that have been postulated to explain the inhibitory effects of lactic acid: (i) toxicity arising from the dissipation of the membrane potential, (ii) acidification of the cytosol, or (iii) intracellular anion accumulation [27].

Given these results, it would be interesting to study *hsp16* expression also upon simultaneous stresses and under conditions affecting the membrane fluidity. Indeed, it was demonstrated that changes in membrane fluidity control the expression of a subset of bacterial sHsps, which are localized in the membrane fraction and, most importantly, can affect membrane physical state and stress tolerance [12,28]. A relevant example of such a biological activity is given by the **sHsps** Lo18 of the lactic bacterium *Oenococcus oeni* [29–31].

## 3. Experimental Section

*L. acidophilus* NCFM was routinely grown at 28 °C in MRS broth at pH 6.8, without shaking. For heat stresses, mid-exponential (OD_600_ = 0.6) cultures were transferred to water baths maintained at 45 °C and 53 °C. Acidic stress was imposed by transferring mid-exponential cultures into MRS broth adjusted to pH 4, with either lactic acid or hydrochloric acid. For bile and salt stresses, *L. acidophilus* cells were harvested by centrifugation (4,500 ×g, 10 min), and re-suspended in 30 mL of fresh MRS broth containing 0.3% (w/v) bile or NaCl (1 M and 2.5 M). Stresses were imposed for 5 min and 15 min.

For real time PCR analysis, total RNAs were extracted using UltraClean Microbial Isolation Kit (MoBio, Carlsbad, CA, USA) according to the manufacturer’s instructions. RNA quality was verified as by gel electrophoresis. About 1 μg of total RNA was retrotranscribed using Quantitect Reverse Trascription (Qiagen, Chatsworth, CA, USA) which includes DNase treatment. Real time PCR was performed on Applied Biosystems 7300 Real-Time PCR System using SYBR Green as fluorescent dye. 5 μL of 20-fold diluted cDNA, was added to 15 μL to real-time PCR Mix containing Power SYBR Green PCR Master Mix (Applied Biosystems, Foster City, CA, USA), and 100 nM of forward and reverse primers for *ldhD* amplification (ldhD forward 5′- GTCGGTGTTGTTGGTACTGG 3′ and ldhD reverse 5′-TTAGCTGGAACGTCTGGTAC-3′), and 250 nM of forward and reverse primers for *hsp16* amplification (*hsp16* forward 5′- CGTGGCCGGTACTAGAAAAG-3′ and *hsp16* reverse 5′- TGCTTTGGTAGGGTGATGGT-3′). Primers sequences were designed using OligoPerfect Designer software (Invitrogen, Carlsbad, CA, USA), secondary structures and dimers formation were controlled using Oligo Analyzer 3.0 software (Integrated DNA Technologies, Coralville, IA, USA). The thermal conditions were as it follows: 95 °C for 10 min followed by 35 cycles of 95 °C for 20 s, 58 °C for 30 s, 72 °C for 30 s. Melting curve analyses were performed to verify the specificity of real-time PCR, by slowly increasing the temperature from 65 °C to 95 °C. All samples were performed in duplicate and a negative control (distilled water) was included in each run. The results were analyzed using the absolute quantification method. The amount of target RNA was determined by running a standard curve obtained with serial dilutions (ratio 1:10) of cloned target gene cDNA. Relative gene expressions were normalized to the quantity of total RNA. The use of *ldhD* gene of *L. acidophilus* as internal control was also assessed.

Sequence comparisons with the GenBank database were accomplished using the National Center for Biotechnology Information (NCBI) BLAST2 [32] network service, with the default parameter values provided. Multiple alignments were performed with the European Bioinformatics Institute (EBI) CLUSTALW2 program [33,34] and visualized using Jalview [35]. The neighbor-joining tree was constructed in ClustalX [33,34] and visualized using TreeView [36].

## 4. Conclusions

The biomedical relevance of *L. acidophilus* is testified by its natural occurrence in the human intestinal microbiota, its probiotic properties, and its possible use as a vaccine delivery system [37–39]. Given its use to drive dairy fermentations and in functional probiotic foods, this species is commonly exposed to multiple physiological stresses. Microbial sHsp not only detain a biotechnological potential in reason of their biochemical properties [40,41], but also find application as biomarkers for preliminary screening of LAB strain technological features [42,43]. Although stress response has been studied extensively in some microrganisms, only a limited number of works deal with *L. acidophilus*. Because of the biomedical and technological relevance of *L. acidophilus*, studies on the stress response mechanisms of this organism would be helpful. Here we characterize, for the first time, the expression pattern of *L. acidophilus hsp16* in relation to stress conditions that this bacterium commonly encounters both in its natural niches and for its diverse biotechnological applications. *L. acidophilus hps16* gene structure, genomic organization, and deduced amino acid sequence were analyzed and compared with other LAB, indicating a strong evolutionary relationship with members of the *Lactobacillus acidophilus* group.

## Figures and Tables

**Table 1.** Small heat shock genes (*shs*) identified so far on the genome of lactic acid bacteria (LAB) and bifidobacteria.

**Organism**	**Genome size (Mb)**	**sHsps number**
*Lactobacillus acidophilus* NCFM	2	1
*Lactobacillus brevis* (strain ATCC 367/JCM 1170)	2.35	1
*Lactobacillus casei* (strain ATCC 334)	2.93	2
*Lactobacillus delbrueckii* subsp. *bulgaricus* (strain ATCC BAA-365)	1.9	1
*Lactobacillus fermentum* IFO 3956	2.1	1
*Lactobacillus gasseri* (strain ATCC 33323/DSM 20243)	1.9	1
*Lactobacillus helveticus* DPC 4571	2.1	1
*Lactobacillus johnsonii* NCC 533	1.83	1
*Lactobacillus plantarum* WCFS1	3.34	3
*Lactobacillus reuteri* (strain ATCC 23272/DSM 20016/F275)	2	1
*Lactobacillus rhamnosus* GG	3	2
*Lactobacillus sakei* subsp. *sakei* (strain 23K)	1.9	1
*Bifidobacterium longum*	2.38	1
*Leuconostoc mesenteroides* subsp. *mesenteroides* ATCC 8293	2.04	1
*Oenococcus oeni* PSU-I	1.8	1
*Pediococcus pentosaceus* ATCC 25745	1.8	1
